# Bone mineral density response prediction following osteoporosis treatment using machine learning to aid personalized therapy

**DOI:** 10.1038/s41598-021-93152-5

**Published:** 2021-07-05

**Authors:** Thiraphat Tanphiriyakun, Sattaya Rojanasthien, Piyapong Khumrin

**Affiliations:** 1 Department of Orthopaedics, Faculty of Medicine, Chiang Mai University, Chiang Mai, 50200 Thailand; 2 Department of Family Medicine, Faculty of Medicine, Chiang Mai University, Chiang Mai, 50200 Thailand; 3 Biomedical Informatics Center, Faculty of Medicine, Chiang Mai University, Chiang Mai, 50200 Thailand

**Keywords:** Osteoporosis, Prognosis

## Abstract

Osteoporosis is a global health problem for ageing populations. The goals of osteoporosis treatment are to improve bone mineral density (BMD) and prevent fractures. One major obstacle that remains a great challenge to achieve the goals is how to select the best treatment regimen for individual patients. We developed a computational model from 8981 clinical variables, including demographic data, diagnoses, laboratory results, medications, and initial BMD results, taken from 10-year period of electronic medical records to predict BMD response after treatment. We trained 7 machine learning models with 13,562 osteoporosis treatment instances [comprising 5080 (37.46%) inadequate treatment responses and 8482 (62.54%) adequate responses] and selected the best model (Random Forests with area under the receiver operating curve of 0.70, accuracy of 0.69, precision of 0.70, and recall of 0.89) to individually predict treatment responses of 11 therapeutic regimens, then selected the best predicted regimen to compare with the actual regimen. The results showed that the average treatment response of the recommended regimens was 9.54% higher than the actual regimens. In summary, our novel approach using a machine learning-based decision support system is capable of predicting BMD response after osteoporosis treatment and personalising the most appropriate treatment regimen for an individual patient.

## Introduction

Osteoporosis is a major health problem, leading to fragility fractures which cause significant mortality in affected patients up to 3–4 times higher than the general population within one year after diagnosis^1–3^. With the growing ageing population, this results in substantially increasing costs to global health-care systems^4^. The goals of osteoporosis treatment are to reduce risk of osteoporotic fractures and improve bone mineral density (BMD), a gold standard tool for diagnosing osteoporosis and assessing response to therapy^5,6^. BMD is also used to assess general health condition and used as a predictor of mortality^7^.

A general strategy for osteoporosis treatment usually begins with oral bisphosphonates as a first-line therapy because of its efficacy, safety, and favorable cost. Nonetheless, physicians still need to subsequently monitor BMD response^8^ and adverse events of the treatment. Focusing on BMD and fracture risk as treatment goals, a goal-directed therapy was proposed to individually select an initial treatment on its probability of reaching expected BMD. By following this treatment approach, physicians are required to regularly assess the risk of fracture during treatment using Fracture Risk Assessment Tool (FRAX)^®^ score and adjust regimens with patient-related factors^9^. To identify the failure of treatment, serial 12–24 month check of lumbar spine BMD and hip or femoral neck BMD are used to monitor the response of osteoporotic treatment. A decrease of less than 3% of lumbar BMD or less than 5% total hip or femoral neck BMD are considered “response” to therapy, while those who have new fractures or a BMD decrease exceeding the aforementioned criteria are considered “inadequate response”^10–12^. However, this optimal approach is not always fully implemented depending on the availability of medications^13^ and the cost of treatment which varies across countries.

Although there is an increasing range of approved therapeutic options; for instance, weak antiresorptive, potent antiresorptive, or anabolic agents that efficiently improve BMD and prevent fractures^8^, one fourth of osteoporosis patients receiving treatment fail to respond with BMD improvement^14,15^. The failure rate is high because the treatment outcome does not only depend on treatment regimens and dosage. There are several other factors that interfere with the treatment outcome, such as BMD before treatment, history of falls, laboratory results, FRAX^®^ score, comorbid conditions, current use of glucocorticoid, secondary osteoporosis, and adhering to treatment^14,16,17^.

Accordingly, modern clinical practice guidelines were adjusted to be more personalized and to precisely choose treatment options based on the risk of 10-year fractures according to country-specific guidelines together with patient lifestyle and nutrition. These strategies seem to help to increase the successful rate of treatment outcomes and are reasonable to apply in clinical practice, but it is hard to find a clear and discrete protocol to make universal decisions because of the uncertainty of clinical adjustment and complex information regarding individual clinical factors and personal history^18–21^. These challenges instigated the idea to consider filling this research gap to find a way of effectively choosing the right choice of initial treatment for an individual patient by taking into account patient characteristics and risk factors as currently there is still no definite approach which is able to identify an appropriate treatment regimen regarding individual treatment outcomes^19^.

Machine learning-based decision support systems is a promising research area which is capable of learning multiple patterns of treatment profiles together with a large number of complex parameters. Application of machine learning has been utilized in the medical field such as a decision support system which recommended a drug of choice that best fit a patient and provided a personalized prediction of therapeutic outcome^22,23^. In the osteoporosis field, machine learning-based solutions have been studied for a while in different aspects including detecting postmenopausal women with osteoporosis risk, and beyond diagnosis purposes, such as identifying osteoporosis patient risk groups using various clinical features^24–27^. Application of machine learning with genomics data have shown an ability to predict BMD and osteoporotic fractures risk^28–30^. In the research area of image analysis, machine learning was applied with magnetic resonance imaging (MRI) and computerized tomography (CT) data and the results showed the capability of screening and predicting osteoporotic fractures^31,32^. However, studies of machine learning with osteoporosis treatment outcomes using BMD information combined with clinical features were still limited.

With the increased use of electronic medical records (EMR), patient data including demographic data, clinical features, hospital visit history, clinical diagnosis, laboratory results, and medication prescriptions were exponentially accumulated and enabled us to research the area utilizing machine learning. Because of the crucial impact in the health-care community and prior successful research outcomes in similar areas, we believe that machine learning-based decision support systems could be potentially leveraged with relevant patient information to inform a new way of improving therapeutic effectiveness in osteoporosis treatment.

In this study, we proposed a personalized osteoporosis treatment approach guided by a machine learning model prediction. We trained machine learning models with previous 10-year osteoporosis treatment profiles and a patient-specific clinical dataset acquired from a real-world database. Then, we leveraged the best machine learning model to develop an automated algorithm which was able to recommend appropriate treatment regimens and dosages, in order to achieve an optimal BMD improvement.

## Materials and methods

This was a retrospective study using EMR dataset collected between January 2010 and December 2019 at a tertiary care teaching hospital serving high-volume osteoporosis treatment services. The study protocol was approved under an ethical approval by the Research Ethics Committee of Faculty of Medicine, Chiang Mai University (Study code: ORT-2562-06764). All methods were carried out with exemption criteria (waiver of informed consent) in accordance with the Research Ethics Committee Faculty of Medicine, Chiang Mai University. All identification data including patient name, surname, address, national identification number, address, phone number, and hospital number were removed. Statistical analysis was performed using R Version 4.0.2 on RStudio Version 1.3.959 (RStudio, Boston, Massachusetts). The data pre-processing and machine learning development steps were performed using Python Programming Language Version 3.8.5 (Python Software Foundation, Wilmington, Delaware). Scikit-learn version 0.23.2 Machine Learning library^33^ was used. All computational processes of the machine learning algorithm were performed on Windows Server 2019, 64-bit Operating System, 8 vCPUs of 2.5 GHz Processor, and 32 GBs of Memory (RAM).

### Data acquisition

**Figure 1:**
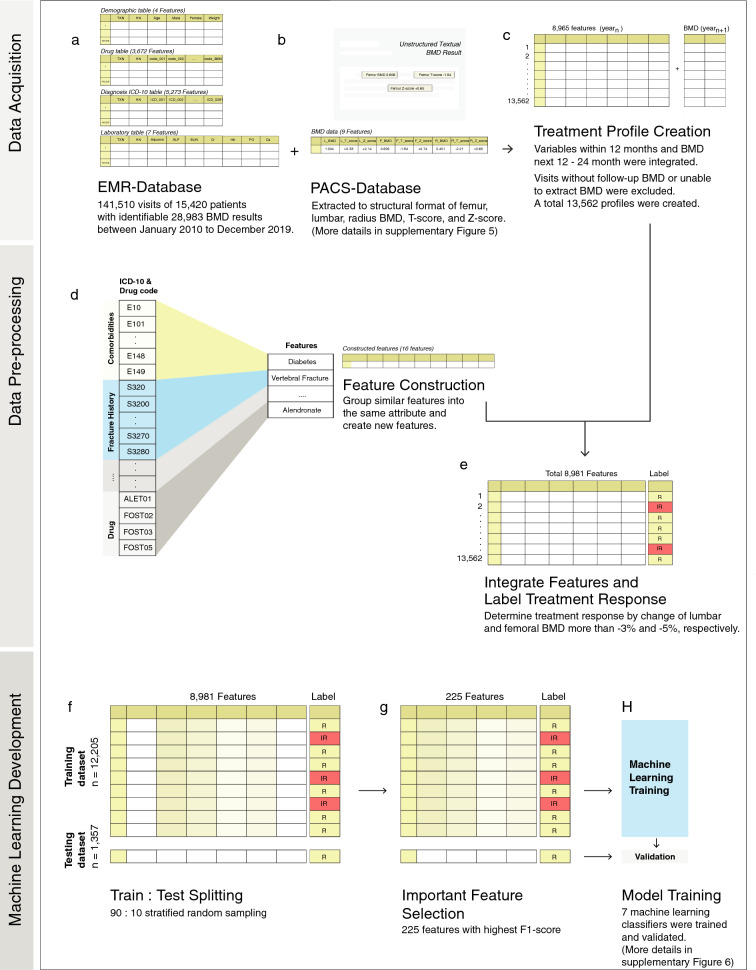
Study protocol to develop machine learning models. (**a**) A high-resolution clinical information was acquired from EMR database. (**b**) Feature construction of ICD10 diagnosis, drug prescription and treatment response labeling. (**c**) Machine learning development process.

A dataset of 141,510 EMR entries from 15,420 patients who had BMD results was acquired from the EMR database All previous International Classification of Disease 10th version (ICD-10) diagnoses, out-patient encounters, in-patient admissions, laboratory results, and medication prescription history in the EMR of enrolled patients were extracted to the dataset, illustrated in Fig. 1a.

The raw datasets were linked by a visit number (TXN), a unique key assigned to a patient on an individual hospital visit or admission. The admission data included visit and admission date (date), gender (categorical values: male or female), age (integer), weight (integer), diagnostic type (categorical values: principal diagnosis or co-morbidities), and ICD-10 codes (categorical values). The drug prescription data included visit/admission date (date) and drug codes (categorical values). The laboratory result data included visit/admission date (date), laboratory items (categorical values), and values (float). All categorical features were split into a one-hot numeric array fashion using the Scikit-learn OneHotEncoder and then grouped by TXN. All codes were additionally mapped with description text for data visualization.

**Figure 2:**
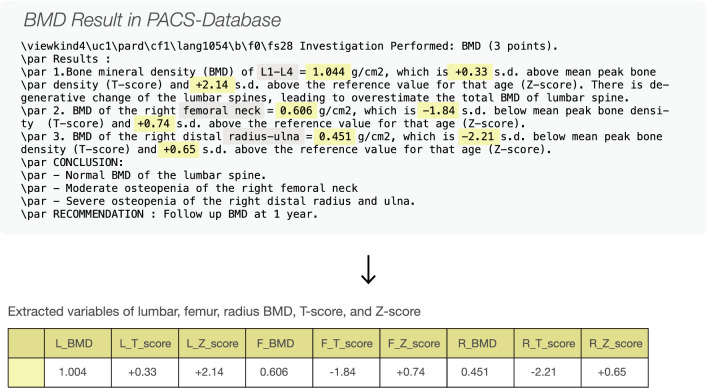
Unstructured pattern of a bone mineral density result in PACS-database was processed using a regular expression search. Lumbar BMD, T-score, Z-score, femur BMD, T-score, Z-score, radius BMD, T-score, and Z-score were extracted, respectively.

We were not able to include some important factors that are associated with risk of fracture^34^ such as height, parental history of hip fracture, current tobacco smoking, daily alcohol consumption of three or more units daily, due to the lack of digital-format data records within our institute. We identified 28,983 BMD results from institute’s PACS database. In our setting, Hologic Horizon^®^ DXA was used as bone densitometer equipment. The results were stored in the database as semi-structured textual format narrated by certified radiologists. The BMD, T-score, and Z-score of femur (hip), lumbar (vertebral), and radius were extracted to structural variables using regular expression search technique. The BMD dataset (Fig. 1b) was consolidated with the patient dataset resulting in the treatment profile dataset (Fig. 1c). Figure 2 shows an example of the BMD features which were extracted from the BMD report.

An individual treatment profile was created by integrating all information between sequential BMD encounters. This protocol excluded visits which had no follow-up BMD or unable to extract BMD results. This strategy was designed to include not only osteoporosis patients (BMD T-score less than $-2.5$) by WHO classification^35^, but also normal (BMD T-score more than $-1.0$) and osteopenia patients (BMD T-score between $-1.0$ and $-2.5$) who were treated by history of fragility fracture, high-risk screening, and followed up patients with increased BMD T-score. A total 13,562 treatment profiles with 8965 categorical and numerical variables were obtained for this study (Fig. 1c).

### Statistical analysis

Baseline characteristics of 13,562 treatment profiles were reported. Continuous variables were described with mean and standard deviation (SD), a Student’s t-test was used to test the differences between response and inadequate response groups of treatment profiles regarding BMD change. Categorical variables were described with count and percentage, and were analyzed as differences of treatment response between groups with Chi-square test, *p* value less than or equal to 0.05 indicating statistical significance.

### Data pre-processing

Within the treatment profile features, we were able to include relevant variables which may affect BMD response^14,16,36^ including Age, Sex, Weight, Previous vertebral fractures and pelvis (ICD10-S32*), Fracture of shoulder and upper arm (ICD10-S42*), Fracture of forearm (ICD10-S52*), Fracture of femur (ICD10-S72*), Fracture of lower leg including ankle (ICD10-S82*), Medical history of Gout (ICD10-M10*), Rheumatoid arthritis (ICD10-M051), Essential (primary) hypertension (ICD10-I10), Diabetes mellitus (ICD10-E1*), Disorders of lipoprotein metabolism and other lipidemias (ICD10-E78*), Heart failure (ICD10-I50), and Ischemic heart disease (ICD10-I2). Previous steroid usage history was collected including Budesonide inhaler, Hydrocortisone injection, oral or intravenous Dexamethasone, and oral Prednisolone. Associated laboratory results including Serum Albumin, Alkaline Phosphatase, Blood Urea Nitrogen, Creatinine, Hemoglobin, Phosphate, and Calcium level were acquired from the database.

Fourteen treatments were identified as features including Calcium (600/835/1500 mg), Vitamin D (Alfacalcidol 0.25/0.5/1 mcg, Ergocalciferol 20,000 IU), Calcitonin (Nasal spray 200 IU/Injection 50 IU), Menatetrenone (5 mg) three times a day, daily oral Raloxifene (60 mg), daily oral Strontium (2 g), weekly oral Alendronate (70 mg with or without Vitamin D), weekly oral Risedronate (35 mg), monthly oral Risedronate (150 mg), monthly oral Ibandronate (150 mg), 3-month intravenous Ibandronate (3 mg), 6-month subcutaneous Denosumab (60 mg), 12-month intravenous Zoledronic acid (5 mg), and daily subcutaneous Teriparatide (20 mcg/dose). Similar comorbidities were grouped into eight new features including Diabetes mellitus (ICD10-E10*), Dyslipidemia (ICD10-E780*), Gout (ICD10-M10*), Vertebral fractures (ICD10-S320*), Fracture of shoulder and upper arm (ICD10-S4200*), Fracture of forearm (ICD10-S520*), Fracture of femur (ICD10-S720*), and Fracture of lower leg including ankle (ICD10-S82*). All anti-osteoporosis agents were grouped by drug generic name to seven features (Calcium, Vitamin D, Calcitonin, Teriparatide, Alendronate, Risedronate, Zolendronate) (Fig. 1d).

We identified outcomes of the model as the next femoral BMD and lumbar BMD change after treatment, from hip and lumbar BMD in continuous variables, respectively (Fig. 1e). Patients who maintained lumbar BMD more than $-3\%$ and femoral BMD more than $-5\%$ from baseline were labeled *“response”* to therapy, whereas patients with BMD loss more than this criteria were labeled to have an *“inadequate response”* to therapy^37^. We simplified the definition of inadequate response to cover the period during 12–24 month follow-up BMD because in practice the BMD follow-up date can vary from 12 to 36 months after treatment^12,18^. Because of this time variation, the treatment outcome may be affected among patients^12,18^.

Finally, the dataset of treatment profiles (8981 variables) contained features of 3672 drug prescriptions, 5273 ICD-10 diagnoses, 7 laboratory results, 4 demographic variables, 9 BMD variables, and 16 newly constructed variables.

### Machine learning development

**Figure 3:**
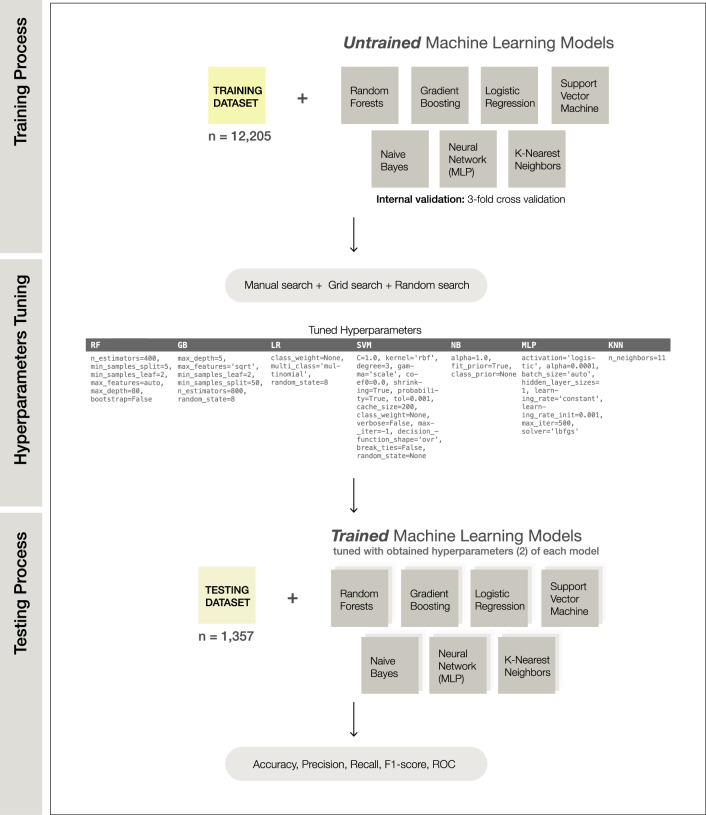
Machine learning development process.

**Figure 4:**
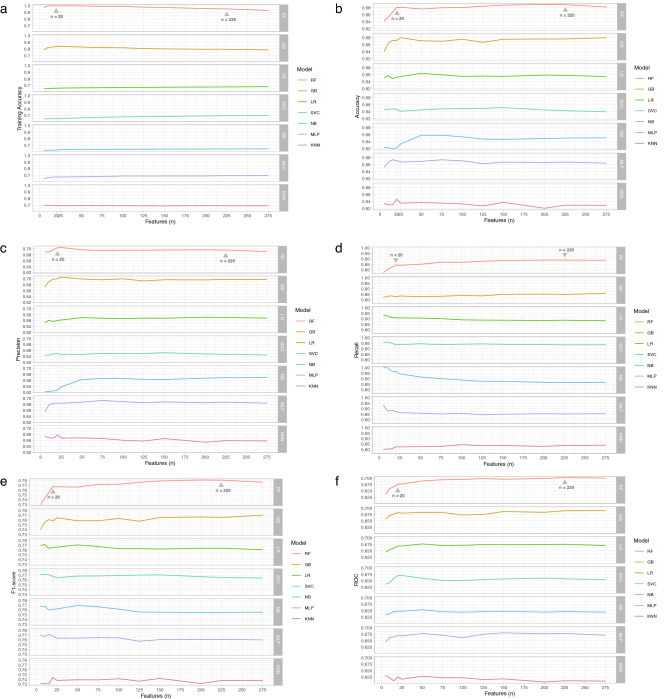
Machine learning performance patterns of different amount of features (5–275 features). (**a**) Accuracy of training dataset. (**b**–**f**) Accuracy, Precision, Recall, F1-score, and AUROC of the testing dataset.

The dataset was randomly partitioned into 90:10 training and testing dataset with stratified random sampling. The training dataset was used to train and adjust parameters by internal cross-validation, while the testing dataset was used to assess the model performance and its generalizability (Figs. 1f, 3). All categorical variables in the dataset were encoded with 0 and 1. Missing values were imputed with mean. All numeric features in the dataset were normalized between 0 to 1 scale and BMD response was set as a labeled output. We implemented a feature selection algorithm (SelectKBest with the score function ANOVA F-value between label/feature for classification tasks) to rank the variables according to highest F1 score to the outcome. We fine-tuned the model examining the number of features from 5 to 8981 during the training and testing process. The results showed that the number of features beyond 20 provided no further significant improvement of the model performance (Fig. 4). However, the number of features at 225 was the best number when considering the highest performance of accuracy, ROC, precision, and recall. Thus, the top 225 features were selected for the model development process. This step reduced the dimension of features in the dataset in order to improve accuracy and reduce computational complexity by removing irrelevant variables (Fig. 1g).

We applied the Shapley (SHAP)^38,39^ and Bayesian inference^40^ to find the top 20th most relevant features and explored how these features influenced the prediction outcome. According to the Bayesian inference interpretation guidelines^40^, we used the pymc3 python package^41^ (version 3.11.2) to calculate 95% Bayesian credible intervals (CrI). We trained the Bayesian logistic regression model with the entire dataset with the top 20th features and reported the result with mean and 2.5th and 97.5th percentile (95% CrI). The guidelines^40^ suggested that if the CrI did not cover zero, this indicated a statistically significant result. Then, we interpreted these features with significant results by describing with the most plausible values (from the lowest to the highest value of CrI) with higher probability of representing the true (unknown) estimate indicating that the mean of the feature of the response group would be different (lower or higher depending on the negative or positive CrI, respectively) compared to the inadequate response group, with at least a 95% probability.

We selected seven machine learning models from the Scikit-learn library^33^ for solving classification problems, consisting of Random Forests Classifier, Gradient Boosting Classifier, Logistic Regression Classifier, Support Vector Machine Classifier, Naive Bayes Classifier, Neural Network (Multilayer Perceptron), and K-Nearest Neighbors Classifier. The manual parameter tuning, grid search, and random search techniques were used to properly obtain the best tuned parameters^42^. The parameters which provided highest accuracy were applied to each algorithm. Accuracy, Precision (Positive Predictive Value), Recall (sensitivity), F1-score, and Area Under Receiver Operating Characteristic curve (ROC) were used to evaluate the performance of each model on the training dataset and testing dataset (Figs. 1h, 3).

## Results

### Exploratory data analysis

The dataset of 13,562 osteoporosis treatment profiles taken from January 2011 to December 2019 were used for this study. The baseline characteristics are shown in Table 1, of which there were 37.46% (5080) entries in the ‘inadequate response to treatment’ group (referred to as the inadequate response group) and 62.54% (8482) entries in the ‘adequate response-group (henceforth referred to as the response group). The average changes of femoral BMD ($-6.09\% \pm 8.24$) and lumbar BMD ($-8.65\% \pm 22.89$) for the inadequate response group were significantly lower than for the response groups (femoral BMD $2.33\% \pm 11.06$ and lumbar BMD $3.80\% \pm 6.83$), respectively. The average age of the total group was $62.23 \pm 10.76$ years old with the average age of the inadequate response group significantly lower than the response group (63.36 and 64.75, respectively)(*p* value $< 0.01$). Co-morbid conditions and basic laboratory results were not different between the two groups, except Alkaline Phosphatase (ALP) which was significantly lower in the response group than in the inadequate group. There were no differences in previous history of hip fractures and vertebral fractures between the groups, whereas forearm fracture history was found significantly more often in the response group than in the inadequate group (*p* value = 0.02).

**Table 1:** Baseline characteristics and clinical features of study population with inadequate response and adequate response of BMD after treatment.

Variables	Total	Inadequate response	Response	*p* value
Treatment profile, n (%)	13,562	5080 (37.46%)	8482 (62.54%)	
Age (mean ± SD) years	62.23 ± 10.76	63.36 ± 10.87	64.75 ± 10.66	*< 0.01*
**Sex, n (%)**				
Women	12,731 (93.87%)	4790 (37.62%)	7941 (62.38%)	0.12
Men	831 (6.13%)	290 (34.90%)	541 (65.10%)	0.12
**Medical history, n (%)**				
Hypertension	483 (3.56%)	182 (37.68%)	301 (62.32%)	0.96
Diabetes	194 (1.43%)	81 (41.75%)	113 (58.25%)	0.24
Dyslipidemia	367 (2.71%)	137 (37.95%)	230 (63.71%)	1.00
Gout	9 (0.07%)	6 (66.67%)	3 (33.33%)	0.14
**Laboratory (mean ± SD)**				
Calcium	9.48 ± 1.46	9.47 ± 0.54	9.48 ± 1.80	0.61
Phosphate	4.00 ± 0.54	4.00 ± 0.21	4.00 ± 0.66	0.57
Albumin	4.30 ± 1.75	4.28 ± 1.27	4.30 ± 1.99	0.43
ALP	69.90 ± 15.95	70.42 ± 19.38	69.58 ± 13.48	*< 0.01*
BUN	14.29 ± 3.16	14.34 ± 3.33	14.26 ± 3.06	0.13
Creatinine	0.98 ± 0.83	0.99 ± 0.85	0.97 ± 0.81	0.26
Hemoglobin	12.24 ± 0.94	12.23 ± 0.97	12.25 ± 0.91	0.24
**Fracture history, n (%)**				
S32* Vertebral fractures	28 (0.21%)	9 (32.14%)	19 (67.86%)	0.70
**Non-vertebral fractures**				
S42* Shoulder and upper arm	25 (0.18%)	9 (36%)	64 (72.73%)	1.00
S52* Forearm	62 (0.46%)	14 (22.58%)	48 (77.42%)	0.02
S72* Hip and Femur	62 (0.46%)	18 (29.03%)	44 (70.97%)	0.21
S82* Lower leg and ankle	16 (0.12%)	8 (50%)	8 (50%)	0.44
**Treatment regimens, n (%)**				
Calcium	10,285 (75.84%)	3776 (36.71%)	6509 (63.29%)	*< 0.01*
Vitamin D	9939 (73.29%)	3558 (35.80%)	6381 (64.20%)	*< 0.01*
Calcitonin	282 (2.08%)	110 (39.01%)	172 (60.99%)	0.63
Menatetrenone	2819 (20.79%)	1091 (38.70%)	1728 (61.30%)	0.13
Anti-osteoporosis drug	7179 (52.93%)	2126 (29.61%)	5053 (70.39%)	*< 0.01*
Weak antiresorptive agents				
Daily oral regimens				
Raloxifene (60 mg)	420 (3.10%)	187 (44.52%)	233 (55.48%)	*< 0.01*
Strontium (2 g)	1188 (8.76%)	313 (26.35%)	875 (73.65%)	*< 0.01*
Potent antiresorptive agents				
Weekly oral regimens				
Alendronate (70 mg)	2383 (17.57%)	649 (27.23%)	1734 (72.77%)	*< 0.01*
Risedronate (35 mg)	1064 (7.85%)	356 (33.46%)	708 (66.55%)	*< 0.01*
Monthly oral regimens				
Risedronate (150 mg)	87 (0.64%)	23 (26.44%)	64 (73.56%)	* 0.04*
Ibandronate (150 mg)	1,711 (12.62%)	529 (41.15%)	1182 (58.85%)	*< 0.01*
Injection regimens				
3-Month Ibandronate (3 mg)	48 (0.35%)	15 (31.25%)	33 (68.75%)	0.46
6-Month Denosumab (60 mg)	176 (1.30%)	32 (18.18%)	144 (81.82%)	*< 0.01*
12-Month Zoledronic acid (5 mg)	809 (5.97%)	218 (26.95%)	591 (73.05%)	*< 0.01*
Anabolic agent				
Injection regimen				
Daily teriparatide (20 mcg/dose)	295 (2.18%)	88 (29.83%)	207 (70.17%)	*< 0.01*
Initial femoral BMD (mean ± SD)	0.61 ± 0.13	0.65 ± 0.14	0.60 ± 0.11	*< 0.01*
Initial lumbar BMD (mean ± SD)	0.79 ± 0.14	0.81 ± 0.15	0.78 ± 0.14	*< 0.01*
Femoral BMD changes (mean ± SD)	$-0.82\% \pm 0.12$	$-6.09\% \pm 8.24$	$2.33\% \pm 11.06$	*< 0.01*
Lumbar BMD changes (mean ± SD)	$-0.87\% \pm 0.18$	$-8.65\% \pm 22.89$	$3.80\% \pm 6.83$	*< 0.01*

*p* value significant at 0.05 level.

Italics font in the "*p* value" column emphasizes the statistical significance at *p* value < 0.01.

#### Distribution of treatment response in the dataset

**Figure 5:**
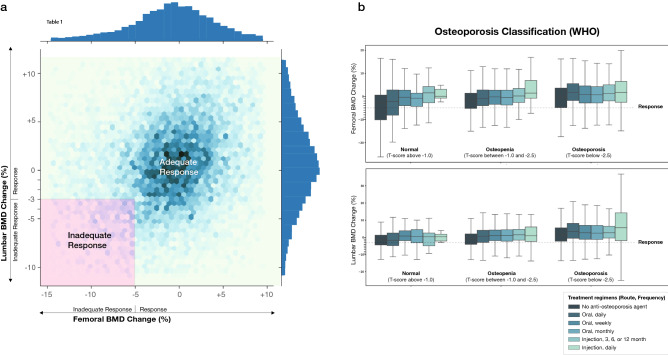
Exploratory data analysis of the dataset. (**a**) The plot was created using lumbar and femoral BMD change after treatment, which consisted of n = 13,562 treatment profiles. The color intensity of hexagon bins represent the density of treatment profiles. (**b**) The boxplot, with outliers removed, of anti-osteoporosis drug prescription and treatment response pattern of normal-BMD, osteopenia, and osteoporosis groups were shown.

Calcium and Vitamin D prescriptions were significantly higher in the response group than the inadequate response group (*p* value $< 0.01$), while Calcitonin and Menatetrenone were not significantly different. The number of treatment profiles receiving anti-osteoporosis agents in the response group (70.39%) was significantly higher than the inadequate response group (29.61%) (*p* value was $< 0.01$). This was true for all anti-osteoporosis agents (*p* value $< 0.05$), except for 3-month Ibandronate (3 mg) with *p* value 0.46. Figure 5a shows the distribution of lumbar and femoral BMD changes after treatment. The majority of treatment profiles responded to treatment (located in the adequate response area). The dataset was described according to the WHO Osteoporosis Classification (Normal, Osteopenia, and Osteoporosis)^35^ as seen in Fig. 5b. The treatment profiles with anti-osteoporosis agents positively contributed to response patterns for both femoral and lumbar BMD, especially in 3, 6, 12-month and daily injection routes.

### Machine learning prediction of treatment response

#### Performance comparison

Seven machine learning models were evaluated for treatment response classification performance using 225 variables with the highest respective F1-scores shown in Table 2. For the training dataset, the accuracy was: Random Forests model = 0.95; Gradient Boosting model = 0.79; Logistic Regression model = 0.68; Support Vector Machine (SVM) model = 0.70; Naive Bayes model = 0.64; Neural Network (Multi Layer Perceptron (MLP)) model = 0.69; and K-Nearest Neighbor model = 0.69. The Random Forests model achieved the highest overall performance in the testing dataset with Accuracy = 0.69, Precision = 0.70, Recall = 0.89, F1-score = 0.78, ROC = 0.70.

**Table 2:** Performance of each classifier model on training and testing dataset on the top 225 features.

Classifier models	Training dataset	Testing dataset				
	Accuracy	Accuracy	Precision	Recall	F1-score	ROC
Random Forests	0.95	0.69	0.70	0.89	0.78	0.70
Gradient Boosting	0.79	0.68	0.70	0.85	0.77	0.69
Logistic Regression	0.68	0.66	0.67	0.89	0.76	0.68
Support Vector Machine	0.70	0.64	0.65	0.94	0.77	0.66
Naive Bayes	0.64	0.65	0.67	0.87	0.75	0.65
Neural Network (MLP)	0.69	0.66	0.68	0.87	0.76	0.67
K-Nearest Neighbors	0.69	0.63	0.66	0.84	0.74	0.61

#### Model selection and interpretation

**Figure 6:**
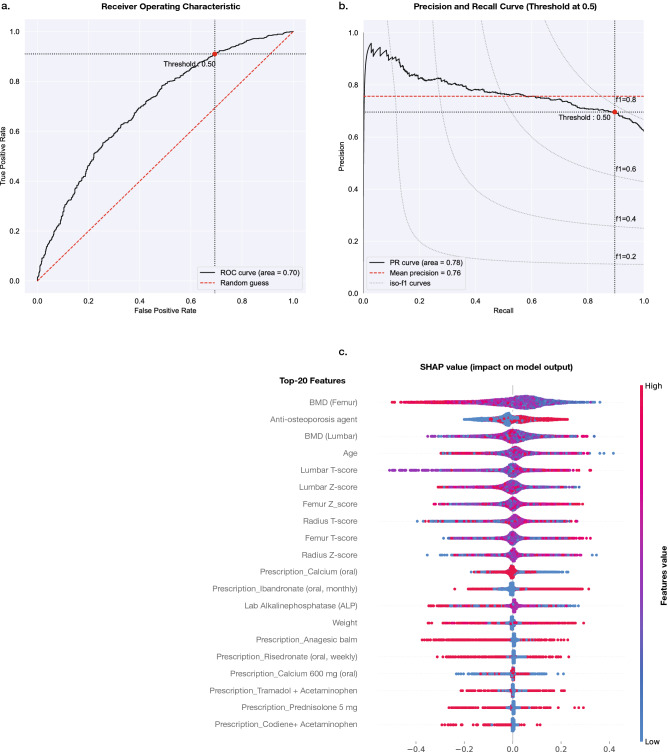
Random Forests classifier model characteristics. (**a**) Reciever-operting characteristic curve (ROC) of the model with AUC of 0.70. (**b**) Precision and Recall (PR) curve for the model achieved AUC of 0.78. (**c**) Variable contribution of the model by SHAP Value. The importance of variables were ranked in descending order. The blue and red color represent low and high value of the variables, respectively.

All seven machine learning models demonstrated a high Recall (Sensitivity) to predict a response to treatment. Among all algorithmic approaches, the Random Forests (RF) model^43^, a tree-based machine learning algorithm, produced the highest accuracy, and positive predictive value (precision), F1-score, and ROC. The final parameters with the best fine-tuned results of all models are presented in Table 3. The value of the area under the receiver operating characteristics curves of Random Forests algorithm and Precision and Recall curve are shown in Fig. 6a,b.

**Table 3:** The best tuned parameters of the models.

Classifier models	Parameters
Random Forests	n_estimators = 400, min_samples_split = 5, min_samples_leaf = 2, max_features = auto, max_depth = 80, bootstrap = False
Gradient Boosting	max_depth = 5, max_features = ‘sqrt’, min_samples_leaf = 2, min_samples_split = 50, n_estimators = 800, random_state = 8
Logistic Regression	class_weight = None, multi_class = ‘multinomial’, random_state = 8, solver=‘lbfgs’, C = 1.0
Support Vector Machine	C = 1.0, kernel = ‘rbf’, degree = 3, gamma = ‘scale’, coef0 = 0.0, shrinking = True, probability = True, tol = 0.001, cache_size = 200, class_weight = None, verbose = False, max_iter = $-1$, decision_function_shape = ‘ovr’, break_ties = False, random_state = None
Naive Bayes	alpha = 1.0, fit_prior = True, class_prior = None
Neural Network (MLP)	activation = ‘logistic’, alpha = 0.0001, batch_size = ‘auto’, hidden_layer_sizes = 1, learning_rate = ‘constant’, learning_rate_init = 0.001, max_iter = 500, solver = ‘lbfgs’
K-Nearest Neighbor	n_neighbors = 11

To interpret feature contributions to the selected model, the Shapley (SHAP)^38,39^ and Bayesian credible interval^40^ were used. SHAP value explanation method was used to rank the 20 most important predictors in Fig. 6. The five most influential variables were: Initial femoral BMD, Anti-osteoporosis agents, Initial lumbar BMD, Age, and Lumbar T-score. Regarding the analysis of Bayesian credible interval, Table 4 shows the mean difference, standard deviation (sd) of the features in the population, CrI, and value interpretation. The High Density Interval (hdi) at 2.5$\mathrm{th}$ (hdi-2.5%) and 97.5$\mathrm{th}$ (hdi-97.5%) represent the lower and higher value of the 95% CrI. Analysis suggests that the most plausible values with higher probability of representing the true (unknown) estimate are the mean difference of the BMD (Femur), age, lumbar Z-score, femur T-score, radius Z-score, Calcium (oral), laboratory result of Alkaline Phosphatase, Analgesic Balm, and Codeine with Acetaminophen, with the response group results significantly lower than for the inadequate response group, with at least a 95% probability. Conversely, the mean difference of anti-osteoporosis agent, lumbar T-score, femur Z-score, radius T-score, Ibandronate (oral, monthly), and weight of the response group are significantly higher than for the inadequate response group, with at least a 95% probability. The mean difference of the other features apart from those just described are not statistically significantly different.

### Individual BMD response prediction

In clinical application, the personalized osteoporosis management approach aims to tailor therapy to individual patients for improving BMD. The proposed machine-learning model identifies possible BMD response patterns of different treatments based on complex personal clinical data.

**Figure 7:**
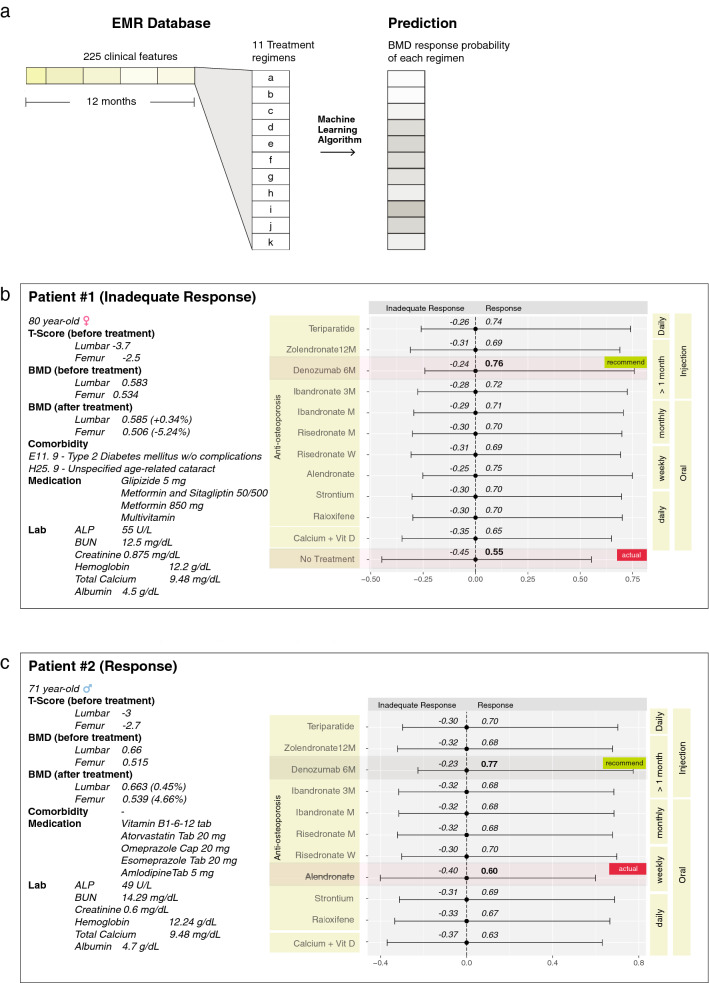
Individual BMD response prediction. (**a**) A set of 225 patient-specfic clinical information within 12 month prior to treatment was combined with an actual regimen and other regimens in order to predict BMD response. (**b**, **c**) The model predict probability of response of each regimen, and recommend the regimen which might provide a highest-probability according to the model prediction.

A set of selected 225 features from the EMR database was given to the computational model with 11 different inputs of anti-osteoporosis treatment regimens (Fig. 7). The output was a patient-specific probability for each treatment outcome. Administration route and frequency of regimens were also labeled. We applied the prediction algorithm to 1357 treatment profiles in the testing dataset. In Table 5, we present the treatment response probability of actual regimens and the recommended regimens. The difference of average probability between actual and recommended regimens were 8.36% in the response group (see an example of a response case in Scenario II), 11.47% in the inadequate response group (see an example of an inadequate response case in Scenario I), and 9.54% in the whole testing dataset.

#### Scenario I: inadequate response to treatment (Fig. 7b)

An 80-year-old female with comorbidities of Diabetes and Cataracts. The initial lumbar and femoral BMD were 0.583 and 0.534, respectively. The lumbar and total femoral BMD T-score were $-3.7$ and $-2.5$, respectively (WHO classification of Osteoporosis^35^). The patient did not receive an anti-osteoporosis agent. At her 1-year follow up, she was considered as having an inadequate response to treatment: her serial lumbar and femoral BMD were 0.585 (+ 0.34%) and 0.506 ($-5.24\%$), respectively. The actual situation showed the lowest predicted probability of response (0.55), while the best recommended regimen (Denozumab 6M) showed 0.76 probability of response. The difference between actual and recommended regimen was + 21%.

#### Scenario II: response to treatment (Fig. 7c)

A 71-year-old male with initial lumbar and femoral BMD of 0.66 and 0.515, respectively. The lumbar and total femoral BMD T-score were $-3$ and $-2.7$, respectively (Osteoporosis according to WHO classification^35^). The patient received an anti-osteoporosis agent (weekly oral Alendronate). At the 1-year follow up, he was considered as having an adequate response to treatment: his serial lumbar and femoral BMD were 0.663 (+ 0.45%) and 0.539 (+ 4.66%), respectively. The algorithm predicted the actual regimen’s response probability of 0.60, while the best recommended regimen (Denozumab 6M) showed 0.77 probability of response. The difference was + 17%.

## Discussion

This study demonstrated the development of a machine learning-based clinical decision support system. The model, which was trained with clinical features, laboratory results, and prescription records from an EMR database, can predict the probability of a BMD response after osteoporosis treatment. This method is an automated data pipeline process which can be potentially integrated into hospital EMR systems. Eventually, it has potential benefits for physicians when selecting therapeutic regimens.

In this study, the majority of patients responded to osteoporosis treatments (see the treatment response in Table 1). However, the percentage of inadequate responses in patients with osteoporosis was relatively high (37.46%) compared with the previous study (25.8%)^14^. Since our dataset was larger and uncontrolled, representing real-world circumstances, this may affect adherence to therapy and response to treatment. In some real-world studies, the adherence rates were reported as low as 25% for one-year treatments^44,45^. This finding of inadequate response confirmed that the poor-compliance issue is a serious problem in our hospital service and also remains a worldwide challenge, and the causes of inadequate response after osteoporosis drug therapy initiation must be explored.

We found that patients in the response group were older than the inadequate response group but baseline femoral and lumbar BMD of the inadequate response group were significantly lower than the response group while the average serum ALP in the inadequate response group was significantly higher than the response group. These results confirm findings by previous research^14^ as relevant factors for predicting the response of osteoporosis treatment. Apart from the relevant factors, another interesting finding is the history of forearm fracture (ICD10-S52).

The number of patients with a history of forearm fractures in the response group was higher than for the inadequate response group (see Table 1). This was the only feature among the history of fractures which was significantly different. This means the patients with a history of forearm fractures had a high successful rate of treatment (77.42%), while the other types of fractures were not different. We manually explored the cause of this finding and found that the average BMD of femur and lumbar ($0.61 \pm 0.13$ and $0.79 \pm 0.14$, respectively) in the forearm fracture group were lower than the average of the population in this study. Because of the low initial BMD before treatment, this group intentionally had a higher chance of receiving anti-osteoporosis treatment. We confirmed this assumption by exploring this patient group and the data showed that 37 of 62 forearm fractures patients (59.68%) received anti-osteoporosis agents which were more than the average of treatment profiles in the population of this study (52.93%). In clinical practice, distal forearm fractures of the wrist are commonly found in the older population who tend to have bone degeneration with high bone loss (low BMD) at the distal forearm^46^. Therefore, the low BMD and high number of treatment resulted in a higher rate of BMD improvement. However, when we explored the treatment response in hip, femur, shoulder, and upper arm fractures, the results showed that the number of the response group was also found more often that the inadequate response group but not at a statistically significant level. We believe that if we could further investigate in a larger and controlled study, we might find some interesting information and significant clinical impact as they are also a common osteoporosis fracture.

For the treatment profiles, we found that Calcium and Vitamin D supplementation were routinely used in the treatment of osteoporosis patients. Calcium and Vitamin D supplementation with/without anti-osteoporosis agents showed significant outcomes from the treatment except in conjunction with 3-month Ibandronate (3 mg) which had a higher number in the response treatment group but not at a statistically significant level, possibly because the sample size was too small.

We observed the major characteristics between the response and inadequate response group recognized by the machine learning model according to the SHAP explanation in Fig. 6c and the CrI interpretation in Table 4 that were relevant to predict response outcome of the treatment. Typical patients in the response group had lower levels of initial femoral and lumbar BMD, received the treatment with anti-osteoporosis agents, and were of a slightly younger age. Meanwhile, the characteristics of the inadequate response group were patients with higher initial femoral BMD, were not treated with anti-osteoporosis agents, and were of a slightly older age compared with the response group.

**Table 4:** Analysis of Bayesian logistic regression of the top 20th features.

Features	Mean	sd	hdi-2.5%	hdi-97.5%	Interpretation
Intercept	4.778	0.648	3.498	5.947	–
BMD (Femur)	− 3.796	0.352	− 4.473	− 3.146	Lower
Anti-osteoporosis agent	0.657	0.048	0.569	0.752	Higher
BMD (Lumbar)	− 0.696	0.547	− 1.745	0.313	Not different
Age	− 0.017	0.003	− 0.023	− 0.013	Lower
Lumbar T-score	0.289	0.069	0.169	0.423	Higher
Lumbar Z-score	− 0.302	0.056	− 0.407	− 0.120	Lower
Femur Z-score	0.554	0.054	0.448	0.649	Higher
Radius T-score	0.133	0.038	0.059	0.201	Higher
Femur T-score	− 0.402	0.057	− 0.509	− 0.295	Lower
Radius Z-score	− 0.105	0.042	− 0.189	− 0.030	Lower
Calcium (oral)	− 0.39	0.073	− 0.524	− 0.253	Lower
Ibandronate (oral, monthly)	0.488	0.065	0.357	0.601	Higher
Lab Alkaline Phosphatase (ALP)	− 0.003	0.001	− 0.005	− 0.002	Lower
Weight	0.003	0.001	0.001	0.005	Higher
Analgesic Balm	− 0.446	0.079	− 0.605	− 0.304	Lower
Risedronate (oral, weekly)	− 0.127	0.08	− 0.278	0.016	Not different
Calcium 600 mg (oral)	0.094	0.065	− 0.023	0.211	Not different
Tramadol + acetaminophen	0.061	0.057	− 0.041	0.172	Not different
Prednisolone 5 mg	− 0.044	0.079	− 0.193	0.097	Not different
Codiene + acetaminophen	− 0.189	0.079	− 0.342	− 0.043	Lower

By the design of this study, we developed a supervised machine learning model using real-world EMR-derived clinical information which consists of elementary variables such as demographic data, co-morbidities, previous diagnosis of fracture, basic laboratory results, and drug prescriptions. These high availability features are basic information stored in almost all EMRs. We assessed the performance of 7 algorithms (Table 2). We decided to use Random Forests algorithm to develop a prediction model according to its overall performance which was slightly higher than the other algorithms. The important step was the feature selection; we compared the performance of machine learning between training with all 8981 features and various amounts of selected features (Fig. 4). We found that using all 8981 features contributed similar or slightly higher performance comparing to lesser amount of features, but the dataset was very large and the model took a much longer time for computational processing. As a result, a smaller set of relevant variables (as lower as 20 features) is more practicable to develop a lightweight machine learning model and to apply in general-setting hospitals where these clinical variables are available.

Feature contributions using SHAP value (in Fig. 6c) revealed that initial BMD results, T-score, and Z-score were potent features to predict response to treatment. However, in clinical practice, some patients with osteoporotic fractures might receive anti-osteoporosis treatment without having a BMD result due to the unavailability of bone densitometry equipment in some remote areas. In that situation, using the general standard guidelines^12,18–21^ might be appropriate for choosing an initial treatment.

This work offers a potential clinical application by recommending a personalized choice of the best anti-osteoporosis regimen based on the prediction of BMD response and clinical factors. In Table 5, a higher probability of response for the recommended regimen was shown compared to the actual regimen that the patient received; this is especially important for inadequate response patients. This additional information could be crucial for physicians to identify low-response osteoporosis patients and choose alternative anti-osteoporosis agents which provide optimal response to the treatment^47^.

**Table 5:** Treatment response prediction of 1357 treatment profiles in the validation set, comparing average response probability of actual regimen and recommended regimen.

Treatment profile	N	Predicted treatment response probability (SD)		
		Actual regimen	Recommended regimen	Difference (%)
Response	845	0.66 (± 0.13)	0.75 (± 0.10)	+ 8.36
Inadequate response	512	0.55 (± 0.16)	0.67 (± 0.13)	+ 11.47
Total	1357	0.62 (± 0.15)	0.72 (± 0.12)	+ 9.54

We demonstrated two scenarios of clinical application that could be further used in a hospital setting. The first scenario showed that Patient 1 (Fig. 7b) had not been prescribed with any anti-osteoporosis agents, resulting in decreased BMD at the 1-year follow up. The algorithm predicted low probability of response in this actual situation (Probability 0.55). However, if the patient had received at least one of the anti-osteoporosis regimens, the treatment outcome would be better compared to the actual situation. The quantification of the predicted outcome difference between actual and alternative regimens may provide evidence to support a physician’s decision to choose the best anti-osteoporosis agent to achieve a desirable outcome. In the case of Patient 2 (Fig. 7c), the patient received an actual regimen of weekly oral Alendronate (predicted response probability 0.60). The model predicted that another anti-osteoporosis treatment would provide a higher probability of response (Probability $> 0.60$). This recommendation would aid a physician to select an alternative better-response treatment, taking into consideration the adhering ability of a different administration protocol such as monthly or 6-monthly treatments to obtain a higher possibility of BMD improvement. However, the application of the model on a recommendation system might need to incorporate awareness of atypical cases. For instance, a patient with co-morbid conditions such as chronic kidney disease might be a contraindication for some anti-osteoporosis drugs. Other special considerations exist, such as risk of atypical femoral fracture, and osteonecrosis of the jaw, for which the prediction model is not able to make an appropriate recommendation.

Machine learning-based prediction models and their application to osteoporosis treatment have shown potential opportunity for widespread adoption, as seen in the FRAX^®^ scenario which has been widely adopted by the World Health Organization (WHO) as indicating standard guidelines to assess individual risk of fracture over 10 years with 12 variables^8,12,18,48^. Although FRAX^®^ has been efficiently used to predict probability of osteoporotic fractures and has been validated in many countries, there are some limitations: physicians need to manually complete a form to calculate the risk on a website and individually assess complex patient information in order to select the treatment of choice. Our proposed model has demonstrated some points of clinical service improvement. First, this machine learning model could be embedded in an automated clinical decision support system in EMR which can be seamlessly implemented to support a clinical practice, without the need for manual variable input. Second, the machine learning development process is scalable and reproducible which enables the model to continuously learn with larger or newer information. Third, the model is applicable to be customized in other institutes to develop a personalized model using their own dataset that suits the characteristics of local patients.

This study has a few notable limitations worth mentioning. First, our prediction model is developed from retrospective information. We were unable to include important missing variables such as weight, height, parental fracture history, smoking history, and alcohol consumption characteristics. These lifestyle features are not routinely recorded in our EMR in a digital format. This missing information might affect the accuracy of the model. Second, this study excluded treatment profiles with no serial BMD which therefore excluded poor-compliance or loss of follow-up patients. As a result, the actual inadequate response group may be higher than in this study and some characteristics of the inadequate response group may not be reflected in the real-world situation. Since the general treatment adherence rate is typically low, even though we included all 10-year treatment profiles in our institute, the number of eligible patients with a long-term follow-up BMD was still limited. Thus, we decided to study only the initial treatment and a single serial BMD, taken 12–24 months after treatment. Third, the current criteria to define inadequate response to osteoporosis treatment is different among different studies^49^. This study complied to our national guidelines to consider BMD decrease as an inadequate response^12^. We determined change of total femoral BMD and lumbar BMD as output variables, despite a femoral neck BMD also being a variable used to evaluate response of treatment. We could not gather the incidence of fracture information after treatment as part of our definition of inadequate response in this study. Lastly, we conducted the experiment in a single-center design which may lead to an over-fitting model; future external validation in other populations is required. Further study should include a cross-institution dataset to broaden the range of the variables, or incorporate genomic information^29^ which may individually affect the response of treatment. We also encourage investigators to create prediction models for long-term treatment outcomes.

## Conclusion

In summary, our results show that it is feasible to use a combination of EMR-derived information to develop a machine learning algorithm to predict a BMD response following osteoporosis treatment. This alternative approach can aid physicians to select an optimal therapeutic regimen in order to maximize a patient-specific treatment outcome.

## Data Availability

De-identified data are available upon reasonable request from qualified investigators.

## Code availability

The code is available upon reasonable request from qualified investigators.
